# Mesenteric extraskeletal osteosarcoma with telangiectatic features: a case report

**DOI:** 10.1186/1471-2407-7-82

**Published:** 2007-05-15

**Authors:** Kyung Hwa Lee, Jae Kyoon Joo, Dong Yi Kim, Ji Shin Lee, Chan Choi, Jae Hyuk Lee

**Affiliations:** 1 Department of Pathology, Chonnam National University Medical School, #8, Hak-dong, Dong-gu, Gwangju, 501-749, Republic of Korea; 2 Division of Gastroenterologic Surgery, Department of Surgery, Chonnam National University Medical School, #8, Hak-dong, Dong-gu, Gwangju, 501-749, Republic of Korea

## Abstract

**Background:**

Extraskeletal osteosarcoma is a rare malignant mesenchymal tumor, with a predominant occurrence in the extremities. Only two cases of mesenteric extraskeletal osteosarcoma have been documented. We describe an unusual case of extraskeletal osteosarcoma with telangiectatic features occurring in the mesentery.

**Case presentation:**

A 67-year-old male presented with blood-tinged stool of 1-month's duration. On colonoscopy, a solid mass was detected protruding from the colon wall. Computed tomography showed a 15 × 9.7 cm heterogeneously enhancing mass, with mottled calcification and a cystic portion, occupying the left upper quadrant of the abdominal cavity. Curative resection of the tumor was performed, and the excised tumor was composed of large multilocular cysts containing old hematomas and necrotic debris. The histology revealed an osteosarcoma showing osteoid formation and blood-filled spaces lined with atypical cells. Despite postoperative chemotherapy, he developed a recurrent peritoneal mass and multiple lung metastases 3 months postoperatively.

**Conclusion:**

Given the rarity of cases of mesenteric extraskeletal osteosarcoma, its biologic behavior at this location remains to be determined. However, extraskeletal osteosarcoma with telangiectatic features is an uncommon entity to be recognized because of the possible fatal outcome related to the tumors.

## Background

Extraskeletal osteosarcoma (EOS) is a rare malignant mesenchymal tumor characterized by the production of neoplastic osseous tissues, without attachment to bone or the periosteum [1]. It occurs predominantly in the lower extremity, upper extremity, and retroperitoneum, and rarely in the visceral organs [2]. Of the different histologic variants of EOS, the telangiectatic variant has only a small number of subtypes, and its pattern is more often mentioned as a minor component of other variants [1-3]. To date, only two cases of extraskeletal osteosarcoma in the mesentery have been reported [4,5]. Here, we describe a case of mesenteric osteosarcoma with telangiectatic features.

## Case presentation

A 67-year-old male presented with abdominal pain and blood-tinged stool of 1-month's duration. His past medical history revealed hypertension well controlled by medication for 8 years and that he had been a smoker for 20 pack years. His family history was noncontributory. Physical examination revealed a protuberant abdomen with a huge tender intra-abdominal mass. Laboratory findings including blood analysis, serum electrolytes, and hepatic and renal functions were within normal limits, as was the serum alkaline phosphatase.

**Figure 1:**
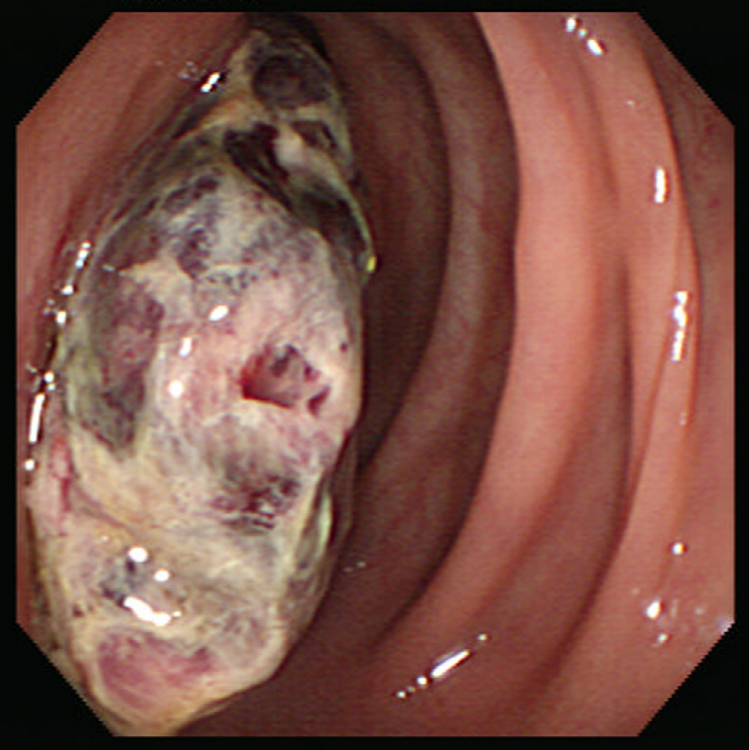
Colonoscopic findings showing a necrotic mass protruding from the colon wall.

**Figure 2:**
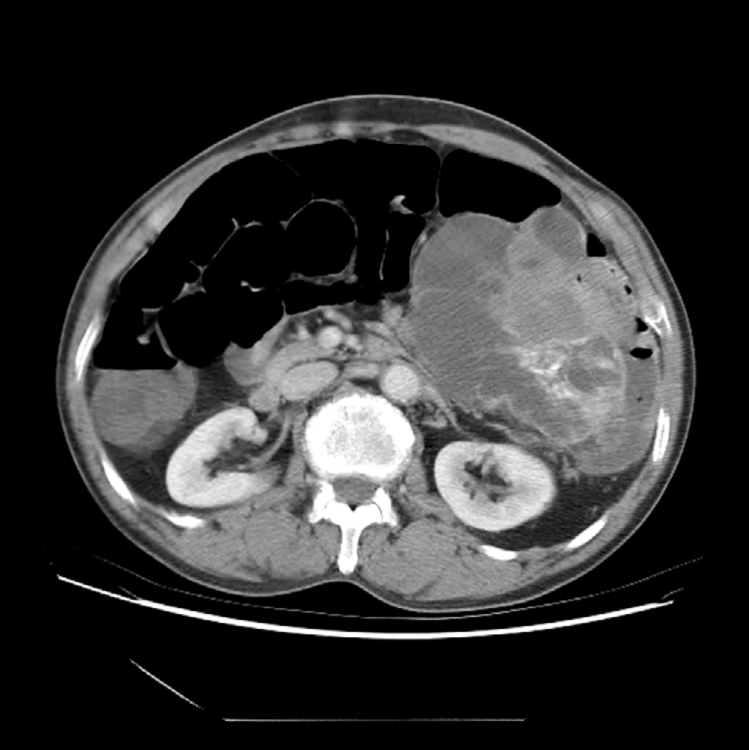
Computed tomography showing a heterogeneously enhancing mass, with mottled calcification and a cystic portion.

**Figure 3:**
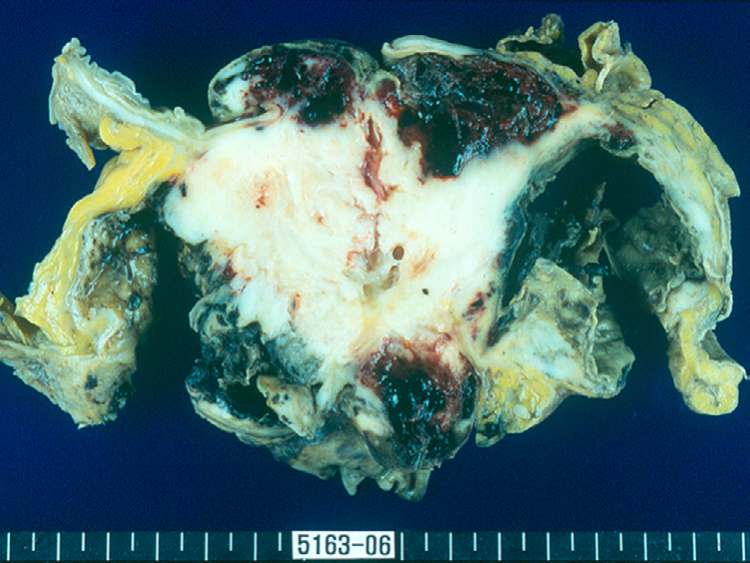
The cut tumor surface composed of a gritty solid area and a collapsed cystic portion containing blood clots.

On colonoscopy, a solid mass measuring 5 cm was detected protruding from the colon wall (Figure 1). The mass was covered with blood clots and necrotic debris. Colonoscopic biopsy confirmed a sarcoma of an undetermined type. Computed tomography demonstrated a 15 × 9.7 cm heterogeneously enhancing mass, with mottled calcification and a cystic portion, occupying the left upper quadrant of the abdominal cavity (Figure 2). For curative resection of the tumor, *en bloc *mass excision with segmental colon resection and splenectomy was performed. During the operation, the surgeon described the tumor as being located in the mesentery and involving the stomach, greater omentum, pancreas, and transverse colon. The resected tumor measured 18.5 × 13 × 9.5 cm. It was located in the mesentery and perforated the abutting colon wall. The external surface of the mass was smooth. The cut surface consisted of a gray-white to tan-yellow solid area with a gritty sensation and a large multicystic area (Figure 3). The cystic portion contained clotted blood with thin septae and was focally necrotic.

**Figure 4:**
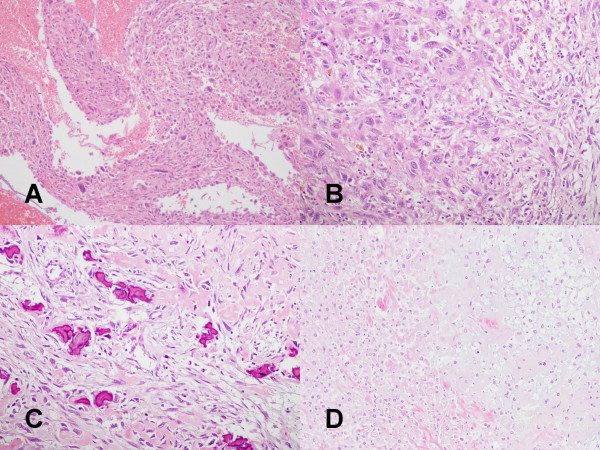
**A**. Telangiectatic foci with several septae composed of large, bizarre cells (×100, H&E). **B**. Poorly differentiated sarcomatous tumor composed of large anaplastic cells (×200, H&E). **C**. Spicules of osteoid and bone between malignant cells (×200, H&E). **D**. Transition areas between loose osteoid and cartilage (×100, H&E).

Sections taken from the cystic structure showed large blood-filled spaces separated by thin septae and smaller cystic spaces within the solid area. The septal walls consisted of numerous large, bizarre rounded to spindled cells with multilobed hyperchromatic nuclei, and coarse granular chromatin (Figure 4A). Sections from the firm, calcified portion revealed a poorly differentiated sarcomatous tumor showing a solid growth pattern with large anaplastic cells and spindle cells (Figure 4B). Giant cells with multiple bizarre nuclei or osteoclast-like giant cells were scattered throughout the tumor and were associated with the areas of hemorrhage. The osteoid was laid down in a fine ramifying lacework, and was partly calcified (Figure 4C). Transition areas between neoplastic osteoid and cartilage were noted (Figure 4D). More than 20 mitoses per ten high-power fields were observed, and these included highly atypical forms. Necrosis was evident.

For immunohistochemical studies, paraffin-embedded tissue was stained using the avidin-biotin peroxidase complex method. The neoplastic cells were positive for vimentin, alpha smooth muscle actin, osteonectin, CD99, and S100 in the chondroblastic portion, but negative for cytokeratin, epithelial membrane antigen, desmin, myogenin, CD34, and c-kit. The final histologic diagnosis was a mesenteric extraskeletal osteosarcoma with telangiectatic features.

After the uneventful surgery, the patient underwent the first cycle of chemotherapy consisting of intravenous ifosfamide (1800 mg/m^2^) and adriamycin (25 mg/m^2^). However, he developed a recurrent peritoneal mass measuring 11 × 9.5 cm and multiple lung and liver metastases 3 months postoperatively. He died 4 months later.

## Conclusion

Extraskeletal osteosarcoma is a rare soft tissue tumor, which is reported to account for approximately 1% of all soft tissue sarcomas and 4% of all osteosarcomas [6,7]. EOS occurs in a patient population distinct from osteosarcoma of bone; EOS most commonly affects individuals older than 30 years and is rarely encountered during the first two decades of life [3,6]. The lower extremity is the most common anatomic site, followed by the upper limb and retroperitoneum [3].

The English literature includes case reports of EOS arising in unusual sites, such as the larynx, kidney, esophagus, small intestine, liver, heart, urinary bladder, parotid, and breast [3]. Osteosarcoma of the mesentery is extremely rare, and only two documented cases exist in the literature. The first case written in German was mentioned briefly in Fine and Stout's review article [5]. That tumor was adherent to the peritoneum and bowel with metastases in mesenteric lymph nodes; the patient, a 39-year-old male, died 55 days postoperatively. Choudur described the second case, a 15-cm tumor in the pelvic cavity of a 45-year-old male without any involvement of the bowel, prostate, or urinary bladder [4]. In our case, the tumor was so large that it was difficult to determine its origin. However, both the preoperative image studies and the intraoperative observation indicated the superficial involvement of multiple intra-abdominal organs and suggested that the tumor centered on the mesentery. These findings strongly suggested that our EOS originated in the mesentery.

Although solid areas in our case showed features of conventional osteosarcoma, the large cystic areas closely resembled telangiectatic EOS. According to Bane *et al*., if the major histologic pattern comprises 75% or more of the tumor, that pattern is considered predominant [3]. Other authors suggested that telangiectatic osteosarcomas (TOSs) should be defined as hemorrhagic, cystic, or necrotic spaces that occupy more than 90% of the lesion [8,9]. Although the large cystic areas in our case accounted for more than 75% of the tumor, they did not amount to 90%. Therefore, the histological diagnosis in our case was extraskeletal osteosarcoma with telangiectatic features. However, current World Health Organization (WHO) classification doesn't present any practical guide on the proportion of cystic portion in diagnosing telangiectatic osteosarcoma [10].

The criteria for the diagnosis of primary extraskeletal osteosarcoma by Allan *et al*. are as follows: the presence of a uniform morphological pattern of sarcomatous tissue that excludes the possibility of malignant mesenchymoma, the production of malignant osteoid or bone by the sarcomatous tissue, and the ready exclusion of an osseous origin [6]. The second criterion of Allan *et al*. is controversial, especially in the telangiectatic variant of osteosarcomas. In an article on telangiectatic osteosarcoma of bone, osteoid was not found in five of 25 cases (25%) [8]. The first detailed report on three cases of telangiectatic EOS also noted the absence of osteoid in one case [1]. Before those reports on the telangiectatic subtype, the diagnosis of osteosarcoma was strictly defined as a mesenchymal tumor characterized by the direct production of osteoid or bone by malignant cells. With the exception of the telangiectatic variant of osteosarcoma, most authors do not advocate the diagnosis of osseous or extraosseous osteosarcoma without the appropriate osteoid production [1,11].

A diligent search for osteoid is very important for the diagnosis of extraosseous osteosarcoma. In our case, the limited amount of tumor tissue in the colonoscopic biopsy concealed the presence of osteoid, so that we were unable to reach a definite diagnosis. In addition, the seemingly contradictory results of the immunohistochemistry were not helpful. Both osseous and extraosseous osteosarcomas show notoriously varied immunophenotypes by reacting to such reagents as factor-XIII related antigen, S100, desmin, alpha-smooth muscle actin, cytokeratin, and epithelial membrane antigen [12,13].

The differential diagnosis includes various benign and malignant soft tissue osteogenic lesions. Previous reports have indicated that many EOS or telangiectatic EOS cases were initially misinterpreted as different entities. Occasionally, EOS has been mistaken for myositis ossificans, which is a benign ossifying process usually occurring in young adults as a solitary, well circumscribed mass within skeletal muscle. The typical lesion of myositis ossificans shows a zoning effect with peripheral differentiation into well formed bone and a lack of cytologic atypia [3]. Tumor osteoid and bone tend to show "reverse" zonation characterized by localization toward the center of the tumor in contrast to that of myositis ossificans. As mesenteric tumors, intraabdominal myositis ossificans, or heterotopic mesenteric ossification (HMO) may show worrisome histologic features, including high cellularity, frequent mitotic figures, cartilage formation, and woven bone production. A distinctly zonated growth pattern without hyperchromatism and bizarre nuclear forms of HMO will be likewise helpful [14]. Other benign processes include giant cell tumor of the soft tissues, hemangioendothelioma, and even blood clots [8]. The lack of anaplasia and rarity of septal formation in the first two should help in the differential diagnosis. Adequate sampling may keep us from making an erroneous diagnosis, such as blood clot, by identifying other tumor components.

The diagnosis of malignant mesenchymoma can be made by showing two or more identifiable malignant elements, exclusive of fibrosarcoma, and chondrosarcoma even when osteogenic sarcoma is identified [3,10]. Among those with a fatty component are dedifferentiated liposarcomas with osseous or cartilaginous elements. A thorough histological review in sufficient sections is needed, as with carcinomatous components in carcinosarcoma. Another differential entity that may be encountered is a pleomorphic malignant fibrous histiocytoma (MFH), which is regarded as synonymous with undifferentiated pleomorphic sarcoma in the current WHO classification [15]. Some authors insist that a close relationship exists between EOS and MFH both microscopically and clinically [2,3]. It has been suggested that if the tumor cells are producing unequivocal osteoid or bone, irrespective of the amount or location, the tumor should be classified as an osteosarcoma, while if the osteoid is doubtful, it should be diagnosed as a pleomorphic MFH or undifferentiated sarcoma [11].

Patients with extraskeletal osteosarcoma generally have a poor prognosis and the majority develops metastatic disease within 3 years of diagnosis [16]. The reported overall mortality rates due to the tumor in the larger series exceed 60% [2,6,7]. Tumor size less than 5 cm seems to be an important prognostic factor for EOSs [3]. In other series, however, small size did not equate with a good prognosis or long-term survival [16]. Given the rarity of cases of telangiectatic EOS or EOS with telangiectatic features in the literature, we agree with previous publications that more information needs to be obtained concerning the clinical outcome for appropriate management, planning, and prognostic estimation [1].

## Competing interests

The author(s) declare that they have no competing interests.

## Authors' contributions

KHL performed the review of the literature and participated in the draft of the manuscript.

JKJ and DYK performed the surgery and provided clinical information of the patient.

CC participated in the histopathological analysis, and in the coordination of the study.

JSL participated in the draft of the study, and in the conception of the study.

JHL conceived the study, was the coordinator of the study, and drafted the manuscript.

All authors read and approved the final manuscript.

## Pre-publication history

The pre-publication history for this paper can be accessed here:



## References

[B1] Mirra JM, Fain JS, Ward WG, Eckardt JJ, Eilber F, Rosen G (1993). Extraskeletal telangiectatic osteosarcoma. Cancer.

[B2] Chung EB, Enzinger FM (1987). Extraskeletal osteosarcoma. Cancer.

[B3] Bane BL, Evans HL, Ro JY, Carrasco CH, Grignon DJ, Benjamin RS, Ayala AG (1990). Extraskeletal osteosarcoma. A clinicopathologic review of 26 cases. Cancer.

[B4] Choudur HN, Munk PL, Nielson TO, Ryan AG (2005). Primary mesenteric extraskeletal osteosarcoma in the pelvic cavity. Skeletal Radiol.

[B5] Fine G, Stout AP (1956). Osteogenic sarcoma of the extraskeletal soft tissues. Cancer.

[B6] Allan CJ, Soule EH (1971). Osteogenic sarcoma of the somatic soft tissues. Clinicopathologic study of 26 cases and review of literature. Cancer.

[B7] Sordillo PP, Hajdu SI, Magill GB, Golbey RB (1983). Extraosseous osteogenic sarcoma. A review of 48 patients. Cancer.

[B8] Matsuno T, Unni KK, McLeod RA, Dahlin DC (1976). Telangiectatic osteogenic sarcoma. Cancer.

[B9] Murphey MD, Robbin MR, McRae GA, Flemming DJ, Temple HT, Kransdorf MJ (1997). The many faces of osteosarcoma. Radiographics.

[B10] Fletcher CD, Unni KK, Mertens F (2002). Pathology and genetics of tumours of soft tissue and bone.

[B11] Kempso RLFC, Evans HL, Hendrickson MR, Sibley RK, Rosai JSL (2001). Cartilaginous and osseous tumors. Tumors of the soft tissues.

[B12] Hasegawa T, Hirose T, Kudo E, Hizawa K, Usui M, Ishii S (1991). Immunophenotypic heterogeneity in osteosarcomas. Human Pathol.

[B13] Lidang Jensen M, Schumacher B, Myhre Jensen O, Steen Nielsen O, Keller J (1998). Extraskeletal osteosarcomas: a clinicopathologic study of 25 cases. Am J Surg Pathol.

[B14] Patel RM, Weiss SW, Folpe AL (2006). Heterotopic mesenteric ossification: a distinctive pseudosarcoma commonly associated with intestinal obstruction. Am J Surg Pathol.

[B15] Fletcher CD (2006). The evolving classification of soft tissue tumours: an update based on the new WHO classification. Histopathology.

[B16] Lee JS, Fetsch JF, Wasdhal DA, Lee BP, Pritchard DJ, Nascimento AG (1995). A review of 40 patients with extraskeletal osteosarcoma. Cancer.

